# Exploring the profound link: Breastfeeding’s impact on alleviating the burden of breast cancer – A review

**DOI:** 10.1097/MD.0000000000037695

**Published:** 2024-04-12

**Authors:** Emmanuel Ifeanyi Obeagu, Getrude Uzoma Obeagu

**Affiliations:** a Department of Medical Laboratory Science, Kampala International University, Kampala, Uganda; b School of Nursing Science, Kampala International University, Kampala, Uganda.

**Keywords:** breast cancer, breastfeeding, cancer, epidemiology

## Abstract

Breastfeeding has emerged as a critical factor in understanding and potentially mitigating the risk of breast cancer among women. This review delves into the intricate relationship between breastfeeding and breast cancer, elucidating the biological mechanisms, protective effects, and broader implications for public health. Epidemiological evidence consistently demonstrates a correlation between breastfeeding and a reduced risk of breast cancer, with longer durations of lactation showing a dose-dependent decrease in risk. The biological nexus between breastfeeding and breast cancer involves hormonal changes and the elimination of potentially damaged cells, influencing breast tissue and potentially mitigating carcinogenesis. Moreover, breastfeeding appears to impact tumor subtypes and aggressiveness, particularly demonstrating associations with lower risks of hormone receptor-negative and certain aggressive breast cancer subtypes. Recognizing the significance of breastfeeding in reducing breast cancer risk has profound public health implications, necessitating comprehensive support, education, and policies to encourage and facilitate breastfeeding.

## 1. Introduction

Breast cancer remains a significant global health concern, affecting millions of women and their families annually.^[1]^ Its multifaceted etiology involves a complex interplay of genetic predisposition, hormonal influences, environmental factors, and lifestyle choices. Amidst this complexity, emerging research has unveiled a profound link between breastfeeding and breast cancer risk reduction, offering a promising avenue for prevention and intervention strategies.^[2]^ The act of breastfeeding, beyond its recognized benefits for infant health and development, has garnered attention for its potential protective effect against breast cancer.^[3]^ Epidemiological investigations spanning diverse populations have consistently highlighted a correlation between breastfeeding and a decreased risk of breast cancer.^[4]^ This connection is not merely coincidental but is grounded in intricate biological mechanisms that influence breast tissue dynamics and hormonal milieu. This paper aims to comprehensively explore the profound link between breastfeeding and breast cancer, elucidating the biological underpinnings, the extent of its protective impact, and the broader implications for public health. Understanding this relationship is pivotal for individuals making informed health choices, healthcare providers offering tailored guidance, and policymakers crafting initiatives to support women’s health.

The intricate biological nexus between breastfeeding and breast cancer involves hormonal alterations during lactation, particularly a decrease in estrogen levels, which might limit the proliferation of breast cells and reduce the risk of malignant transformations.^[5]^ Additionally, the cyclical shedding of breast tissue during breastfeeding may eliminate potentially damaged cells, contributing to a decreased likelihood of carcinogenesis. Moreover, investigations suggest that breastfeeding duration plays a crucial role, revealing a dose–response relationship wherein longer periods of breastfeeding confer a more substantial risk reduction.^[6]^ Furthermore, beyond lowering overall breast cancer risk, breastfeeding appears to exert nuanced effects on tumor biology, showing associations with reduced risks of certain aggressive breast cancer subtypes, such as hormone receptor-negative and triple-negative breast cancers.

Recognizing the significance of breastfeeding in reducing breast cancer risk carries profound implications for public health. Encouraging and facilitating breastfeeding initiatives become integral not only for infant health but also for women’s long-term well-being. However, societal and cultural barriers, workplace constraints, and inadequate support systems often impede breastfeeding initiation and sustainability, highlighting the need for comprehensive strategies that address these challenges. In light of the growing body of evidence supporting the link between breastfeeding and breast cancer risk reduction, this review aims to consolidate current knowledge, identify existing gaps, and emphasize the necessity of fostering supportive environments and policies that promote breastfeeding. Addressing these facets can potentially alleviate the burden of breast cancer, paving the way for holistic approaches toward women’s health and well-being on a global scale.

## 2. Breastfeeding

Breastfeeding, recognized as a cornerstone of early childhood development, is a natural and invaluable process wherein a mother provides essential nutrition and immunological protection to her newborn through breast milk. It is a dynamic and multifaceted interaction between mother and child that offers numerous health benefits for both.^[7]^ The composition of breast milk is remarkably intricate, adapting to the evolving needs of the infant. It contains a balance of nutrients, antibodies, enzymes, and growth factors crucial for optimal growth and development. Colostrum, the initial milk produced after childbirth, is particularly rich in antibodies, providing the newborn with essential immune support. As breastfeeding progresses, breast milk composition adjusts to meet the changing nutritional requirements of the growing infant.^[8]^

Beyond its nutritional aspects, breastfeeding plays a pivotal role in establishing a strong emotional bond between mother and child. The physical closeness and skin-to-skin contact during breastfeeding promote a sense of security and emotional connection, fostering the baby’s overall well-being.^[9]^ Moreover, breastfeeding confers numerous health benefits for both the mother and the infant. For infants, breast milk serves as a protective shield against infections, reducing the risk of gastrointestinal illnesses, respiratory infections, allergies, and chronic diseases later in life. Studies also suggest that breastfed babies might have a lower risk of obesity and certain developmental issues.^[10]^

For mothers, breastfeeding offers a myriad of advantages. It aids in postpartum recovery by facilitating uterine contractions and reducing the risk of excessive bleeding. Additionally, breastfeeding contributes to the mother’s long-term health, potentially reducing the risk of ovarian and breast cancers, as well as osteoporosis.^[11]^ The World Health Organization and various health authorities globally strongly advocate for exclusive breastfeeding during the first 6 months of a baby’s life, followed by continued breastfeeding alongside appropriate complementary foods up to 2 years of age or beyond.^[12]^

However, despite the established benefits, breastfeeding rates vary across regions and communities. Several factors, including societal norms, lack of support, employment constraints, insufficient knowledge, and medical conditions, can pose challenges to breastfeeding initiation and duration. Efforts to promote breastfeeding involve creating supportive environments that facilitate and encourage breastfeeding. This includes implementing breastfeeding-friendly workplace policies, providing education and support to mothers and families, training healthcare professionals to offer breastfeeding guidance, and fostering a societal culture that values and supports breastfeeding mothers.^[13]^

Ultimately, breastfeeding stands not only as a fundamental aspect of infant health and development but also as a contributor to maternal well-being and the overall health of families and communities. Recognizing its significance and addressing barriers to breastfeeding are crucial steps toward fostering a healthier future for both mothers and children.

## 3. Breast cancer

Breast cancer is a prevalent malignancy characterized by the abnormal growth of cells in the breast tissue. It is one of the most common cancers affecting women worldwide and, although less common, can also occur in men.^[14]^ The development of breast cancer is influenced by various factors, including genetic predisposition, hormonal influences, lifestyle choices, and environmental exposures. Mutations in specific genes, such as BRCA1 and BRCA2, are associated with an increased risk of developing breast cancer, although most cases are not directly linked to inherited genetic mutations.^[15,16]^

The disease manifests through various forms, ranging from noninvasive types like ductal carcinoma in situ to invasive types that spread beyond the original site. Commonly, breast cancer is categorized into different subtypes based on the presence or absence of specific receptors on the tumor cells, such as estrogen receptors (ER), progesterone receptors (PR), and human epidermal growth factor receptor 2 (HER2). These subtypes influence treatment decisions and prognosis.^[17]^

Early detection plays a critical role in managing breast cancer. Screening methods, including mammography, clinical breast examinations, and self-exams, aid in identifying abnormalities in the breast tissue at an early stage, facilitating prompt diagnosis and treatment.^[18]^ The symptoms of breast cancer can vary, but common signs include the presence of a lump or thickening in the breast or underarm area, changes in breast size or shape, nipple abnormalities such as inversion, redness or scaling of the breast skin, and nipple discharge other than breast milk. However, it is essential to note that these symptoms can also occur due to benign conditions.^[19]^

Treatment approaches for breast cancer depend on several factors, including the type and stage of cancer, its characteristics, and the patient’s overall health. Treatments may include surgery (such as lumpectomy or mastectomy), radiation therapy, chemotherapy, hormone therapy, targeted therapy (for tumors expressing specific receptors like HER2), and immunotherapy. Personalized treatment plans, often involving a combination of these modalities, are designed to achieve the best possible outcome for each patient.^[20]^ Preventive measures and risk reduction strategies for breast cancer include maintaining a healthy lifestyle, avoiding excessive alcohol consumption, maintaining a healthy weight, being physically active, and, in some cases, considering preventive surgeries for individuals at high risk due to genetic predisposition.^[21]^ Research into breast cancer continues to advance, exploring new treatment modalities, precision medicine approaches, and understanding the underlying biology of the disease. Additionally, awareness campaigns, education, and support networks play a vital role in empowering individuals to understand the risks, seek timely screenings, and navigate the challenges associated with a breast cancer diagnosis.

## 4. The biological nexus

The biological nexus between breastfeeding and breast cancer represents a multifaceted interplay of hormonal, physiological, and cellular mechanisms that contribute to the observed associations between these 2 phenomena.

### 4.1. Hormonal influence

One of the primary factors contributing to the link between breastfeeding and reduced breast cancer risk involves hormonal changes that occur during lactation.^[22]^ Prolactin, a hormone produced during breastfeeding, stimulates milk production and concurrently suppresses ovulation. This suppression of ovulation leads to reduced exposure to estrogen, a hormone known to promote the growth of certain types of breast cancer cells. Lower levels of estrogen during breastfeeding may contribute to a decreased risk of breast cancer by limiting the proliferation of breast cells, thereby reducing the potential for malignant transformation. Additionally, the hormone oxytocin, released during breastfeeding, plays a role in milk ejection and has been associated with potential antitumor effects. Oxytocin may influence cell growth and differentiation in breast tissue, potentially contributing to the protective effect against breast cancer.

### 4.2. Cellular dynamics

Breastfeeding-induced changes in breast tissue dynamics also contribute to the biological nexus.^[23]^ The process of lactation involves cyclical changes in breast tissue, including milk production, ductal expansion, and subsequent involution (returning to a non-lactating state). This cycle of breast tissue remodeling and involution may eliminate potentially damaged or mutated cells, effectively clearing them from the breast tissue. This process of tissue turnover during lactation might reduce the risk of malignant transformation by clearing cells that could otherwise progress to cancer.

### 4.3. Immunological factors

Breast milk contains a variety of immune factors, including antibodies, immune cells, and cytokines, which provide crucial protection to the infant against infections. These immunological components may also influence the breast tissue environment in lactating women. They could potentially contribute to immune surveillance, aiding in the identification and elimination of abnormal or potentially cancerous cells within the breast tissue.^[24]^

Furthermore, breastfeeding might have systemic effects on the immune system of the mother, potentially modulating inflammation and immune responses that could impact cancer development. While these biological mechanisms suggest a plausible association between breastfeeding and reduced breast cancer risk, the exact mechanisms and their relative contributions to cancer risk reduction remain subjects of ongoing research. The complex interplay of hormonal, cellular, and immunological factors underscore the multifaceted nature of this relationship, highlighting the need for further exploration and understanding of the intricate biological nexus between breastfeeding and breast cancer.

## 5. The protective effect

The protective effect of breastfeeding against breast cancer has been extensively studied and documented in epidemiological research. Numerous studies have consistently demonstrated that breastfeeding plays a significant role in reducing the risk of developing breast cancer, offering a notable protective effect to women.^[25]^ One of the key factors influencing the protective effect of breastfeeding is its duration and intensity. Studies have shown that longer durations of breastfeeding are associated with a more substantial reduction in breast cancer risk. The cumulative effect of breastfeeding over time appears to confer greater protection. Moreover, exclusive breastfeeding (feeding the infant only breast milk without any supplemental formula or solid food) has been suggested to potentially offer additional protective benefits compared to mixed feeding.^[26,27]^

Research findings often indicate a dose-response relationship between breastfeeding and breast cancer risk reduction. Each additional month or year of breastfeeding has been associated with a modest but incremental decrease in the risk of developing breast cancer. This suggests that even small increments in breastfeeding duration may contribute to lowering the risk of the disease.^[28,29]^ The protective effect of breastfeeding against breast cancer has been observed across diverse populations, transcending geographical, racial, and ethnic boundaries. Studies conducted in various countries and among different ethnic groups consistently show a reduced risk of breast cancer among women who have breastfed compared to those who have not.^[30–32]^ The protective effect of breastfeeding appears to have a more pronounced impact on certain subtypes of breast cancer. Research indicates a stronger risk reduction in hormone receptor-positive breast cancers, particularly among postmenopausal women. These types of breast cancers, which are influenced by hormonal factors like estrogen and progesterone, show a decreased risk among women with a history of breastfeeding.^[31,32]^

Overall, the cumulative evidence strongly supports the notion that breastfeeding exerts a protective effect against breast cancer. This protective effect is not only influenced by the duration and intensity of breastfeeding but also extends to specific subtypes of breast cancer. Understanding the nuances of this protective effect aids in emphasizing the importance of breastfeeding as a potentially modifiable factor in reducing the risk of breast cancer and underscores the significance of promoting and supporting breastfeeding initiatives for the health and well-being of women.

## 6. Impact on subtypes and aggressiveness

Breastfeeding’s impact on the subtypes and aggressiveness of breast cancer has garnered attention in research due to its potential influence on modifying the characteristics of breast tumors. Studies suggest that breastfeeding may have varying effects on different subtypes of breast cancer and could potentially affect tumor aggressiveness.

### 6.1. Hormone receptor-negative breast cancer

One area of interest lies in the relationship between breastfeeding and hormone receptor-negative breast cancers, including triple-negative breast cancer (TNBC).^[33]^ Hormone receptor-negative breast cancers lack ER, PR, and HER2 expression. These subtypes tend to be more aggressive and have limited treatment options compared to hormone receptor-positive breast cancers. Research findings indicate a potential association between breastfeeding and a reduced risk of hormone receptor-negative breast cancers.^[30–32]^ Several studies suggest that a history of breastfeeding, particularly for an extended duration, may confer a more pronounced protective effect against hormone receptor-negative tumors. The exact biological mechanisms underlying this relationship are still being explored, but the association between breastfeeding and a lower risk of these aggressive subtypes is a significant area of interest in breast cancer research.

### 6.2. Triple-negative breast cancer

TNBC, a subtype characterized by the absence of ER, PR, and HER2 expression, poses challenges in treatment due to the lack of targeted therapies typically effective against hormone receptor-positive or HER2-positive breast cancers.^[34]^ Studies investigating the association between breastfeeding and TNBC risk have shown mixed results. While some studies suggest a potential protective effect of breastfeeding against TNBC, others have not found a significant association. Further research is needed to elucidate the precise impact of breastfeeding on TNBC development and aggressiveness.^[35,36]^

### 6.3. Impact on tumor characteristics

In addition to subtype-specific effects, there is emerging evidence suggesting that breastfeeding may influence certain tumor characteristics. Some studies have suggested that women with a history of breastfeeding may have tumors with less aggressive features, such as smaller tumor size, lower grade, and a reduced likelihood of lymph node involvement.^[37,38]^ These findings imply that breastfeeding might contribute to modifying the biological characteristics of breast tumors, potentially affecting their aggressiveness and clinical behavior.

## 7. Public health implications

The profound link between breastfeeding and reducing the risk of breast cancer holds significant public health implications, emphasizing the importance of promoting, supporting, and advocating for breastfeeding initiatives at various levels of society. Recognizing the role of breastfeeding in breast cancer prevention has implications that extend beyond individual health to broader public health strategies.

### 7.1. Breastfeeding promotion for women’s health

Encouraging and facilitating breastfeeding aligns with public health initiatives aimed at enhancing women’s health and well-being. Promoting breastfeeding as a modifiable factor in reducing breast cancer risk empowers women to make informed decisions about their health. Accessible information and support networks can help women understand the potential long-term benefits of breastfeeding not only for their infants but also for their own health, including breast cancer risk reduction.

### 7.2. Breastfeeding-friendly policies and environments

Developing breastfeeding-friendly policies in healthcare, workplaces, and communities is crucial. Healthcare providers play a pivotal role in educating and supporting women regarding breastfeeding initiation, techniques, and continued support. Workplace policies that accommodate breastfeeding mothers by providing lactation support programs, designated nursing areas, flexible schedules, and parental leave contribute to sustained breastfeeding durations.

### 7.3. Community support and education

Raising awareness about the protective effect of breastfeeding against breast cancer within communities is essential. Education campaigns targeting diverse populations can dispel myths, address cultural barriers, and promote positive attitudes towards breastfeeding. Peer support groups and community programs can offer practical guidance and emotional support to breastfeeding mothers, fostering a supportive environment conducive to sustained breastfeeding.

### 7.4. Inclusion in public health strategies

Incorporating breastfeeding promotion and support into public health agendas strengthens efforts aimed at preventing chronic diseases, including breast cancer. Integrating breastfeeding advocacy into broader health policies not only addresses breast cancer risk reduction but also aligns with goals related to maternal and child health, reducing healthcare costs, and promoting healthier communities overall.

### 7.5. Research and policy advocacy

Continued research into the relationship between breastfeeding and breast cancer is vital for a more comprehensive understanding of this connection. Advocacy efforts focused on policies that prioritize breastfeeding support, allocate resources for research, and promote evidence-based practices are essential. Engaging policymakers to enact supportive legislation and funding research initiatives can further strengthen public health strategies related to breastfeeding and breast cancer prevention.

## 8. Challenges and future directions

Despite the established benefits of breastfeeding in reducing the risk of breast cancer, several challenges persist, hindering widespread adoption and sustained breastfeeding practices. Addressing these challenges and charting future directions are essential for maximizing the potential impact of breastfeeding on breast cancer prevention and overall women’s health. Sociocultural norms, perceptions, and beliefs surrounding breastfeeding vary across different societies and cultures. Stigma, misconceptions, and lack of social acceptance can pose significant barriers to breastfeeding initiation and duration. Efforts to challenge cultural norms and promote positive attitudes towards breastfeeding through education and awareness campaigns are crucial.^[39]^ Workplace limitations, inadequate support systems, and the absence of breastfeeding-friendly environments can impede a woman’s ability to initiate and continue breastfeeding. Insufficient maternity leave, lack of designated lactation spaces, and societal pressures to return to work may hinder breastfeeding. Implementing supportive workplace policies, extending parental leave, and offering workplace lactation support programs are imperative to facilitate continued breastfeeding.^[40]^

Healthcare providers’ knowledge, attitudes, and support practices regarding breastfeeding play a pivotal role in influencing women’s decisions and experiences. Enhancing healthcare provider training on lactation support, breastfeeding counseling, and addressing breastfeeding challenges can improve the overall support provided to breastfeeding mothers. Disparities in breastfeeding rates and access to support disproportionately affect marginalized communities, underserved populations, and low-income families. Tailored interventions addressing socioeconomic inequalities, providing equitable access to breastfeeding education, support services, and lactation resources are essential to bridge these gaps and promote breastfeeding inclusivity.^[41]^ Continued research is necessary to deepen our understanding of the mechanisms underlying the protective effect of breastfeeding against breast cancer, especially in diverse populations and across different breast cancer subtypes. Longitudinal studies investigating the impact of breastfeeding duration, exclusivity, and patterns on breast cancer risk are essential. Disseminating evidence-based information through educational programs and public health campaigns can empower women with accurate knowledge to make informed choices regarding breastfeeding. Advocacy for policy changes that support breastfeeding-friendly environments, enact legislation for paid parental leave, and prioritize lactation support programs is critical. Collaborative efforts involving policymakers, healthcare professionals, advocacy groups, and community stakeholders are essential to drive policy changes that promote and protect breastfeeding rights.^[42]^

## 9. Recommendations

Develop and implement educational campaigns targeting expectant mothers, families, healthcare providers, and communities. These programs should emphasize the long-term benefits of breastfeeding for both infant and maternal health, including its role in reducing breast cancer risk. Healthcare providers play a crucial role in supporting breastfeeding. Enhance training programs for healthcare professionals to provide evidence-based, empathetic, and nonjudgmental breastfeeding counseling and support. Encourage routine discussions about breastfeeding during prenatal and postnatal care visits. Advocate for policies that support breastfeeding in the workplace, such as extended maternity leave, flexible work hours, designated lactation rooms, and breastfeeding breaks. Encourage employers to implement supportive measures to facilitate continued breastfeeding for working mothers. Establish and expand community-based support groups, lactation consultants, peer counseling programs, and hotlines. These initiatives create networks where breastfeeding mothers can seek guidance, share experiences, and receive encouragement from peers and trained professionals. Address cultural barriers and misconceptions about breastfeeding through culturally sensitive educational materials and community outreach programs. Respect diverse cultural practices while promoting the benefits of breastfeeding and encouraging supportive environments for all mothers. Advocate for policies that protect and promote breastfeeding rights, such as laws supporting breastfeeding in public spaces, provisions for breastfeeding accommodations, and mandates for breastfeeding-friendly healthcare facilities.

Invest in research initiatives focused on elucidating the specific mechanisms underlying the protective effect of breastfeeding against breast cancer. Support longitudinal studies examining the impact of breastfeeding duration, exclusivity, and patterns on breast cancer risk across diverse populations. Sustain public health campaigns to raise awareness, disseminate evidence-based information, and encourage societal support for breastfeeding. Utilize various media channels to reach a wide audience and debunk myths surrounding breastfeeding. Collaborate with community leaders, policymakers, advocacy groups, and healthcare organizations to create a collective effort towards promoting breastfeeding-friendly environments and policies. Implement systems to evaluate the effectiveness of breastfeeding support programs and policies. Regularly monitor breastfeeding rates, maternal satisfaction, and the impact on breast cancer incidence to inform ongoing improvements and interventions.

## 10. Conclusion

The profound link between breastfeeding and its potential impact on reducing the burden of breast cancer represents a critical intersection of maternal and women’s health. Extensive research has highlighted the protective effect of breastfeeding against breast cancer, shedding light on its multifaceted biological mechanisms, dose-dependent risk reduction, and potential influence on tumor subtypes and aggressiveness. Recognizing breastfeeding as a modifiable factor in reducing breast cancer risk underscores the importance of promoting, supporting, and advocating for breastfeeding initiatives across various sectors of society. Empowering women with accurate information, fostering supportive environments, and addressing societal, workplace, and cultural barriers are paramount in encouraging breastfeeding initiation and sustaining breastfeeding durations.

The implications extend beyond individual health choices to broader public health strategies. Promoting breastfeeding not only benefits maternal and infant health but also aligns with global health goals, contributing to disease prevention, health equity, and community well-being. By fostering a collective commitment across healthcare systems, workplaces, communities, and policymakers, societies can move towards creating supportive environments that enable women to make informed choices about breastfeeding, potentially reducing the global burden of breast cancer and advancing the health and well-being of women worldwide. Ultimately, recognizing and supporting breastfeeding as a fundamental element of women’s health can pave the way for comprehensive strategies aimed at promoting a healthier future for generations to come.

## Author contributions

**Conceptualization:** Emmanuel Ifeanyi Obeagu.

**Methodology:** Emmanuel Ifeanyi Obeagu.

**Supervision:** Emmanuel Ifeanyi Obeagu.

**Visualization:** Emmanuel Ifeanyi Obeagu, Getrude Uzoma Obeagu.

**Writing – original draft:** Emmanuel Ifeanyi Obeagu, Getrude Uzoma Obeagu.

**Writing – review & editing:** Emmanuel Ifeanyi Obeagu, Getrude Uzoma Obeagu.

## References

[1] Ginsburg O Bray F Coleman MP. The global burden of women’s cancers: a grand challenge in global health. Lancet (London, England). 2017;389:847–60. 27814965 10.1016/S0140-6736(16)31392-7 PMC6191029

[2] Zhong AX Chen Y Chen PL. BRCA1 the versatile defender: molecular to environmental perspectives. Int J Mol Sci . 2023;24:14276. 37762577 10.3390/ijms241814276 PMC10532398

[3] Kim KU Kim WH Jeong CH. More than nutrition: therapeutic potential of breast milk-derived exosomes in cancer. Int J Mol Sci . 2020;21:7327. 33023062 10.3390/ijms21197327 PMC7582863

[4] Roheel A Khan A Anwar F. Global epidemiology of breast cancer based on risk factors: a systematic review. Front Oncol. 2023;13:1240098. 37886170 10.3389/fonc.2023.1240098 PMC10598331

[5] Lee S Kelleher SL. Biological underpinnings of breastfeeding challenges: the role of genetics, diet, and environment on lactation physiology. Am J Physiol Endocrinol Metab. 2016;311:E405–22. 27354238 10.1152/ajpendo.00495.2015 PMC5005964

[6] Gallay C Meylan P Mermoud S. Genetic predisposition and environmental factors associated with the development of atopic dermatitis in infancy: a prospective birth cohort study. Eur J Pediatr. 2020;179:1367–77. 32144501 10.1007/s00431-020-03616-5

[7] Modak A Ronghe V Gomase KP. The psychological benefits of breastfeeding: fostering maternal well-being and child development. Cureus. 2023;15:e46730. 38021634 10.7759/cureus.46730 PMC10631302

[8] Martin CR Ling PR Blackburn GL. Review of infant feeding: key features of breast milk and infant formula. Nutrients. 2016;8:279. 27187450 10.3390/nu8050279 PMC4882692

[9] Krol KM Grossmann T. Psychological effects of breastfeeding on children and mothers. Bundesgesundheitsblatt Gesundheitsforschung Gesundheitsschutz. 2018;61:977–85. 29934681 10.1007/s00103-018-2769-0 PMC6096620

[10] Lyons KE Ryan CA Dempsey EM. Breast milk, a source of beneficial microbes and associated benefits for infant health. Nutrients. 2020;12:1039. 32283875 10.3390/nu12041039 PMC7231147

[11] Ciampo LA Ciampo IR. Breastfeeding and the benefits of lactation for women’s health. Revista Brasileira de Ginecologia e Obstetrícia. 2018;40:354–9. 29980160 10.1055/s-0038-1657766 PMC10798271

[12] World Health Organization. Guideline: counselling of women to improve breastfeeding practices. World Health Organization; 2018. 30933442

[13] World Health Organization. Guideline: protecting, promoting and supporting breastfeeding in facilities providing maternity and newborn services. World Health Organization; 2017. 29565522

[14] Obeagu EI Babar Q Vincent CC. Therapeutic targets in breast cancer signaling: a review. J Pharm Res Int. 2021;33:82–99.

[15] Obeagu EI Babar Q Vincent CC. Advances in therapeutic strategies of immunotherapy in cancer treatment. World J Pharm Pharm Sci. 2021;10:2144–64.

[16] Aizaz M Khan M Khan FI. Burden of breast cancer: developing countries perspective. Int J Innovative Applied Res. 2023;11:31–7.

[17] Makki J. Diversity of breast carcinoma: histological subtypes and clinical relevance. Clinical Med Insights. 2015;8:CPath.S31563. 10.4137/CPath.S31563 PMC4689326 26740749

[18] Bonilla JM Tabanera MT Mendoza LR. Breast cancer in the 21st century: from early detection to new therapies. Radiología (English Edition). 2017;59:368–79. 10.1016/j.rx.2017.06.003 28712528

[19] Ikhuoria EB Bach C. Introduction to breast carcinogenesis–symptoms, risks factors, treatment and management. Eur J Eng Technol Res. 2018;3:58–66.

[20] Waks AG Winer EP. Breast cancer treatment: a review. JAMA. 2019;321:288–300. 30667505 10.1001/jama.2018.19323

[21] Britt KL Cuzick J Phillips KA. Key steps for effective breast cancer prevention. Nat Rev Cancer. 2020;20:417–36. 32528185 10.1038/s41568-020-0266-x

[22] Franca-Botelho AD Ferreira MC Franca JL. Breastfeeding and its relationship with reduction of breast cancer: a review. Asian Pac J Cancer Prev. 2012;13:5327–32. 23317179

[23] Hazra A Bose P Sunita P. Molecular epigenetic dynamics in breast carcinogenesis. Arch Pharm Res. 2021;44:741–63. 34392501 10.1007/s12272-021-01348-0

[24] Lokossou GA Kouakanou L Schumacher A. Human breast milk: from food to active immune response with disease protection in infants and mothers. Front Immunol. 2022;13:849012. 35450064 10.3389/fimmu.2022.849012 PMC9016618

[25] Anstey EH Shoemaker ML Barrera CM. Breastfeeding and breast cancer risk reduction: implications for black mothers. Am J Prev Med. 2017;53:S40–6. 28818244 10.1016/j.amepre.2017.04.024 PMC6069526

[26] Yang Z Ding Y Song S. Factors affecting the breastfeeding duration of infants and young children in China: a cross-sectional study. Nutrients. 2023;15:1353. 36986082 10.3390/nu15061353 PMC10051738

[27] Pathirana MM Ali A Lassi ZS. Protective influence of breastfeeding on cardiovascular risk factors in women with previous gestational diabetes mellitus and their children: a systematic review and meta-analysis. J Hum Lact. 2022;38:501–12. 34609211 10.1177/08903344211034779

[28] Unar-Munguía M Torres-Mejía G Colchero MA. Breastfeeding mode and risk of breast cancer: a dose–response meta-analysis. J Hum Lact. 2017;33:422–34. 28196329 10.1177/0890334416683676

[29] Su Q Sun X Zhu L. Breastfeeding and the risk of childhood cancer: a systematic review and dose–response meta-analysis. BMC Med. 2021;19:1–23. 33845843 10.1186/s12916-021-01950-5 PMC8042913

[30] Agurs-Collins T Dunn BK Browne D. Epidemiology of health disparities in relation to the biology of estrogen receptor–negative breast cancer. Semin Oncol. 2010;37:384–401. 20816508 10.1053/j.seminoncol.2010.05.002

[31] Ramani VK Naik R. A narrative review of the association between pesticides, or-ganochlorines and breast cancer: current advances and research perspectives. Int Arch Public Health Community Med. 2020;4:049.

[32] Perez-Rodriguez J de La Fuente A. Now is the time for a postracial medicine: biomedical research, the National Institutes of Health, and the perpetuation of scientific racism. Am J Bioeth. 2017;17:36–47. 10.1080/15265161.2017.1353165 28829268

[33] John EM Hines LM Phipps AI. Reproductive history, breast-feeding and risk of triple negative breast cancer: the Breast Cancer Etiology in Minorities (BEM) study. Int J Cancer. 2018;142:2273–85. 29330856 10.1002/ijc.31258 PMC5893409

[34] Lehmann BD Pietenpol JA Tan AR. Triple-negative breast cancer: molecular subtypes and new targets for therapy. Am Soc Clin Oncol Educ Book. 2015;35:e31–9. 10.14694/EdBook_AM.2015.35.e31 25993190

[35] Medina MA Oza G Sharma A. Triple-negative breast cancer: a review of conventional and advanced therapeutic strategies. Int J Environ Res Public Health. 2020;17:2078. 32245065 10.3390/ijerph17062078 PMC7143295

[36] Kumari L Mishra L Patel P. Emerging targeted therapeutic strategies for the treatment of triple-negative breast cancer. J Drug Target. 2023;31:889–907. 37539789 10.1080/1061186X.2023.2245579

[37] Peterson MS Gegios AR Elezaby MA. Breast Imaging and Intervention during Pregnancy and Lactation. Radiographics. 2023;43:e230014. 37708073 10.1148/rg.230014 PMC10560982

[38] Chung HL Joiner J Ferreira Dalla Pria HR. Breast imaging considerations in symptomatic young, pregnant, and lactating women. Current Breast Cancer Reports. 2023;15:119–26.

[39] Joseph FI Earland J. A qualitative exploration of the sociocultural determinants of exclusive breastfeeding practices among rural mothers, North West Nigeria. Int Breastfeeding J. 2019;14:1–11. 10.1186/s13006-019-0231-z PMC6701117 31452669

[40] Vilar-Compte M Hernández-Cordero S Ancira-Moreno M. Breastfeeding at the workplace: a systematic review of interventions to improve workplace environments to facilitate breastfeeding among working women. Int J Equity Health. 2021;20:1–21. 33926471 10.1186/s12939-021-01432-3 PMC8082937

[41] Reis-Reilly H Fuller-Sankofa N Tibbs C. Breastfeeding in the community: addressing disparities through policy, systems, and environmental changes interventions. J Hum Lact. 2018;34:262–71. 29596763 10.1177/0890334418759055 PMC6377056

[42] Hernández-Cordero S Pérez-Escamilla R Zambrano P. Countries’ experiences scaling up national breastfeeding, protection, promotion and support programmes: comparative case studies analysis. Matern Child Nutr. 2022;18:e13358. 35438250 10.1111/mcn.13358 PMC9113475

